# Postoperative care in conflict-affected northwest Syria: a mixed-methods study assessing the perspectives of patients, health workers, and non-governmental organizations

**DOI:** 10.1186/s13031-026-00802-4

**Published:** 2026-05-22

**Authors:** Camila Polinori, Maia C. Tarnas, Kelli Wagner, Mohammad Darwish, Stefany M. Lazieh, Ahmad Alnasser, Ahmad Ghandour, Ismail Alkhatib, Saverio Bellizzi, Bara Zuhaili, Gilbert Burnham

**Affiliations:** 1 Johns Hopkins Bloomberg School of Public Health, Baltimore, Maryland USA; 2 Department of Population Health and Disease Prevention, University of California Irvine, California, USA; 3 Johns Hopkins University School of Medicine, Baltimore, Maryland USA; 4 Darkoush General Hospital, Idlib, Syria; 5 Department of Health Policy and Global Health, Ankara Yildirim Beyazit University, Ankara, Turkey; 6 University of Sassari, Sassari, Italy; 7 National Guard Hospital, Madina, Saudi Arabia

**Keywords:** Syria, Conflict, Postoperative care, Health system

## Abstract

**Introduction:**

Thirteen years of conflict have profoundly disrupted Syria’s health system. In northwest Syria, constraints such as deliberate attacks on healthcare, chronic underfunding, and a fragmented health system have created a fragile surgical ecosystem in which health workers operate under extreme resource constraints, patients struggle to access care, and postoperative complications are increasingly likely. In this study, we aim to assess the burden of postoperative complications by examining patient-reported outcomes and barriers to care while integrating this data with insights from health workers and non-governmental organization (NGO) stakeholders.

**Methods:**

This mixed-methods study uses secondary data from a cross-sectional survey of 261 adult patients who underwent surgery in hospitals in northwest Syria. Firth’s logistic regression was used to estimate adjusted odds of complications by surgery type. To contextualize these findings, a workshop engaged 31 surgeons, hospital administrators, and surgeons to identify perceived drivers of complications and prioritize interventions across the WHO health system building blocks.

**Results:**

Barriers to seeking postoperative follow-up care were reported by 57.5% of participants, with transportation, cost, appointment availability, and proximity among the most prevalent barriers. Trauma and orthopedic surgeries demonstrated notably higher odds of adverse outcomes. Workshop participants identified workforce shortages, surgeon rotation across hospitals, fragmented health information systems, and underfunded systems as factors perceived to be associated with these challenges. Workshop participants proposed interventions focused on expanding clinical training, strengthening infection prevention and control, improving provider–patient communication, and establishing governance mechanisms that incentivize quality care.

**Conclusions:**

Our findings illustrate how systemic vulnerabilities shaped outcomes for surgical patients and the surgical ecosystem in northwest Syria. Although data collection occurred during the Assad regime, the findings provide relevant insights for Syria’s health system reconstruction, as addressing the identified challenges will be critical not only for strengthening the surgical ecosystem but also for supporting long-term recovery and resilience efforts.

## Introduction

For over 13 years, the Syrian conflict profoundly fractured the country’s health system [1].

The conflict, which began in 2011, has been characterized by systematic and deliberate attacks on healthcare, widespread displacement, chronic underfunding of humanitarian aid, and the politicized delivery of health services. Healthcare attacks have included airstrikes, arrests and assaults on health workers, and interference with health services [2]. As of 2024, only 54% of hospitals and 39% of health centers were fully functional [3]. Moreover, approximately 70% of Syria’s health workforce has fled the country, creating significant gaps in service provision and an overburdened workforce that faces increased pressure and stress [4]. These vulnerabilities are exacerbated by worsening socioeconomic conditions marked by high inflation, currency depreciation, and fuel shortages [5].

Before the fall of the Assad regime in December 2024, parallel government structures across Syria and the rise of subnational health systems made health service delivery more difficult. In northwest Syria, a region previously under opposition control that experienced large-scale internal displacement and violence [6], these challenges have been particularly severe. The health system relied heavily on humanitarian aid and non-governmental organizations (NGOs) to provide care in the region, including to over 3.4 million internally displaced people (IDPs) [7].

The conflict has also caused a significant rise in conflict-related injuries which often require immediate surgical intervention [8]. In this context, it is difficult to meet care standards when treating such wounds (including those from heavy explosive devices and that are heavily contaminated with shrapnel), which creates markedly higher infection risk [8]. The surgical ecosystem in northwest Syria has been further impacted by the interruption of medical supplies and shortages of essential surgical equipment and medicines [2–8]. Concurrently, individuals are often unable to access care due to high costs, transportation challenges, and long distances to health facilities [2].

These constraints have created a fragile and fragmented surgical ecosystem in which health workers operate under extreme resource limitations, patients face barriers to accessing timely care, and postoperative complications are more likely to occur. Despite this, there is limited research focused on assessing postoperative outcomes and follow-up care in northwest Syria. A study on peripheral extremity surgery in northern Syria cited the loss of electricity and limited surgical equipment as challenges that impacted surgical outcomes [8]. Work by Lazieh et al. on postoperative outcomes in the region has similarly highlighted systemic constraints on surgical care [9]. However, the few studies that do exist have largely focused on clinically observed outcomes, injury patterns, or surgical activity, with limited reporting on post-operative complications, system-level conditions, and barriers to follow-up care. Additionally, existing studies have not sufficiently incorporated observations from key stakeholders and health workers. To address these gaps, our study assesses the burden of postoperative complications by examining patient-reported outcomes and barriers to care, while integrating these data with insights from health workers and NGO stakeholders involved in delivering and managing surgical services in northwest Syria. While this study was conducted during the Assad regime, the findings offer insights into how systemic disruptions have translated into tangible challenges for surgical patients and the surgical ecosystem. These findings are relevant to Syria’s transition as efforts to rebuild the health system must address remaining structural vulnerabilities.

## Methods

### Setting and study design

The study used a convergent mixed-methods design to examine postoperative care in conflict-affected northwest Syria, which, during the study period, consisted of parts of Idlib and Aleppo governorates outside government control. To facilitate and conduct study activities, we partnered with Horizons for Humanitarian Relief Organization (AFAQ), a non-profit organization that provides humanitarian aid to Syrians. The study drew on two complementary data sources: a patient survey and a two-day in-person workshop with hospital administrators, surgeons, and NGO stakeholders. Quantitative and qualitative data strands were analyzed separately and then integrated during analysis to examine how patient-reported postoperative complications and barriers to care aligned with health workers’ and NGO stakeholders’ perspectives on health system factors shaping these outcomes.

#### Survey

The research team received secondary, de-identified data from a cross-sectional telephone survey conducted by AFAQ with 261 patients who had undergone surgery in northwest Syria. A total of 1,893 patients were eligible for inclusion in the sampling frame, defined as adults aged 18 years or older who underwent surgery in participating hospitals in northwest Syria between January 1 and March 31, 2023. From this pool, 261 patients were selected using simple random sampling, which represents 13.8% of all eligible patients. The goal of the survey was to assess surgical complications experienced by patients and barriers to accessing follow-up care. AFAQ developed the survey with surgeons to ensure questions captured the range of possible complications. The survey used the Clavien-Dindo classification system, which is a standardized method for documenting post-surgical complications that has been widely validated across diverse surgical disciplines [10]. Because this study relied on secondary survey data, we did not have access to recruitment flow information, including the number of patients who were unreachable by phone or refused participation, as these records were not retained in the shared dataset. Data collection occurred in a highly unstable setting shaped by conflict, displacement, the February 2023 earthquake and its aftermath, and operational changes among humanitarian organizations, which may have affected recruitment documentation and follow-up.

All participating hospitals were managed by NGOs and shared patient contact information directly with AFAQ to conduct the survey. Two native Arabic speakers reviewed the survey to ensure accuracy and appropriate terminology. AFAQ trained a team of Arabic-speaking data collectors using a ten-hour online training module to standardize data collection. Data collected included surgery type, postoperative complications, barriers to accessing follow-up care, self-reported health status, and any follow-up care with a surgeon or other clinician. Participant data was handled by AFAQ in accordance with strict ethical safeguards. Data collectors were also trained to use non-technical descriptions and probing questions to reduce misclassification. The research team did not have access to any identifiable patient information as data were anonymized before being shared for analysis.

#### Workshop

Following the survey, a two-day in-person workshop was held in Gaziantep, Türkiye. The first session included 6 h of dialogue with 13 participants from NGOs in northwest Syria that were directly responsible for the provision or management of surgical services. The second session also included six hours of dialogue with 18 hospital administrators and surgeons working at facilities in northwest Syria. The inclusion of surgeons, hospital administrators, and NGO stakeholders was intended to capture insights across multiple levels of the health system to comprehensively identify drivers of postoperative complications and opportunities for system-wide improvement. The workshop complemented the survey findings by situating patient-reported complications and barriers to care within the broader health system context, particularly regarding referral gaps, limited follow-up capacity, workforce and supply constraints, and weaknesses in providing continuity of care.

Sessions explored potential factors perceived to be associated with postoperative complications and barriers to implementing solutions. During both sessions, participants identified potential solutions to improve postoperative care in northwest Syria and categorized each according to the six WHO health system building blocks [11].Workshop discussions were conducted without prior exposure to the quantitative patient survey findings; participants drew exclusively on their own professional experience and contextual knowledge to identify drivers of complications and propose interventions. Survey data were analyzed separately and integrated only at the synthesis stage, in keeping with the convergent mixed-methods design of the study. Workshop participants then independently rated each proposed solution on a scale from 1 (worst) to 5 (best) for five criteria: impact, feasibility, affordability, efficiency, and timeliness; these criteria were drawn from the WHO’s systems thinking framework for health system strengthening [11]. Participants were briefed before scoring that each criterion should be assessed on its own merits relative to the intervention in question. This structured scoring approach is consistent with multi-criteria decision analysis (MCDA), a systematic priority-setting method used in health system planning to evaluate competing interventions across multiple performance dimensions simultaneously. Final priority scores were calculated by first averaging individual ratings across all respondents for each criterion, then taking the mean of those criterion-level averages to produce an overall score per intervention, as detailed in the Workshop Analysis section below.

### Study participants

The survey included patients who had undergone surgery in northwest Syria between January 1 and March 31, 2023, were at least 18 years old, spoke Arabic at a conversational level, and were reachable via phone within the first 90 days after surgery. Patients were recruited via simple random sampling from a list of all eligible patients. Oral consent was collected before beginning the survey.

NGO stakeholders were purposively selected to participate in the first workshop session based on their responsibility for the management or funding of hospital operations in northwest Syria. Representatives from the Syrian American Medical Society, Union of Medical Care and Relief Organizations, Syria Relief and Development, Relief International, Horizons for Humanitarian Relief Organization, Al-Sham Humanitarian Foundation, Alameen Organization, Hand in Hand for Aid and Development, and Independent Doctors Association participated in the workshop session. For the second workshop session, invitations were sent to hospitals in northwest Syria that provided surgical services. A total of 18 participants representing hospitals (as surgeons or administrators) took part in the second workshop session. Because dual practice is widespread and legally permitted in Syria, many surgeons worked across multiple hospitals.

### Statistical analysis

Descriptive statistics were used to summarize patient outcomes, complications, and barriers to accessing follow-up care. Complications included fever, nutritional deficits requiring total parenteral nutrition (TPN), wound infection, nausea or vomiting, additional surgery, blood transfusion(s), pneumonia, abscess, admission to the intensive care unit, respiratory failure, deep vein thrombosis, dialysis or kidney failure, and death. For relevant surgeries, complications also included joint dysfunction and bone nonunion. The amount of missing data was low across variables included in the descriptive and regression analyses.

Firth’s logistic regression was used to assess the association between surgery type and the six most commonly reported complications (fever, TPN, wound infection, nausea or vomiting, additional surgery, and blood transfusion), adjusted for hospital-level factors. This method was used because it applies a penalized likelihood estimation to reduce small-sample bias. Confidence intervals (CI) were calculated using the profile method. We restricted complication-specific models to the six most frequently reported complications because they provided sufficient event counts for stable estimation. For each surgery type, individual models were run with each complication as the response variable, as were models measuring the odds of experiencing any complication (i.e., at least one complication) and multiple complications (i.e., at least two complications). Odds ratios (OR) and 95% CIs were reported for each complication type. Regressions were run for all surgery types that had at least ten patients. For this reason, we did not produce models for vascular (*n* = 1 patient), neonatal or pediatric (*n* = 4), ophthalmological (*n* = 1), spine (*n* = 1), or unknown (*n* = 2) surgeries. All quantitative analyses were conducted in R, version 4.4.2.

### Workshop analysis

Workshop notes and facilitator documentation were analyzed using a thematic approach, employing the six WHO health system building blocks as the analytical framework for coding. Four analysts reviewed the workshop material, applied codes, and discussed emerging themes. As workshop participants had not been exposed to the patient survey results prior to or during the sessions, qualitative themes and proposed interventions were generated independently of the quantitative findings and subsequently integrated during the convergent analysis. In each of the six pillars, we analyzed responses thematically and by role (i.e., surgeon, hospital administrator, or NGO stakeholders). The scores for each proposed intervention were averaged across all respondents for each criterion. We also took the mean of all scores across all criteria to calculate an overall score for each intervention. Workshop notes and facilitator documentation were analyzed manually.

## Results

### Survey results

Among the 261 survey respondents, the distribution of surgeries was: general (47.1%, 123/261), orthopedic (23.8%, 62/261), obstetrics and gynecology (14.9%, 39/261), urology (10%, 26/261), and trauma (8.4%, 22/261) (Table 1). Twenty-five patients underwent concurrent surgeries or had surgeries that fit in two categories. Most respondents rated their post-surgery health positively, with 42.9% (112/261) reporting the best possible health rating (5 out of 5) and 34.9% (91/261) rating their health as a 4. Only 1.1% (3/261) of participants rated their post-surgery health as 1 (worst), and 1.1% (3/261) were reported deceased by family members when contacted.

A majority (70.9%, 185/261) of patients reported receiving follow-up care from a surgeon, while 35.6% (93/261) consulted other health professionals. Follow-up rates varied by surgery type, with orthopedic (88.7%, 55/62) and trauma (86.4%, 19/22) surgery patients seeking follow-up care from a surgeon most often. The lowest rate was in obstetrics and gynecology surgeries, with 38.5% (15/39) of patients seeking follow-up care.

The mean number of complications was 1.7 per patient (range 0–8), and 38.3% (100/261) of patients reported multiple complications. The most commonly reported complications were fever (26.8%, 70/261), nutritional deficits requiring the use of TPN (22.2%, 58/261), wound infection (19.5%, 51/261), nausea or vomiting (16.5%, 43/261), additional surgery (13.8%, 36/261), blood transfusion (13.4%, 35/261), and pneumonia (11.1%, 29/261). For relevant surgeries, complications included joint dysfunction (45.6%, 31/68) and bone nonunion (20.6%, 14/68). These findings were reinforced by workshop discussions, in which participants described system-level challenges, including inconsistent postoperative follow-up, limited supply availability, and fragmented referral processes, that may contribute to the persistence of complications.

**Table 1:**
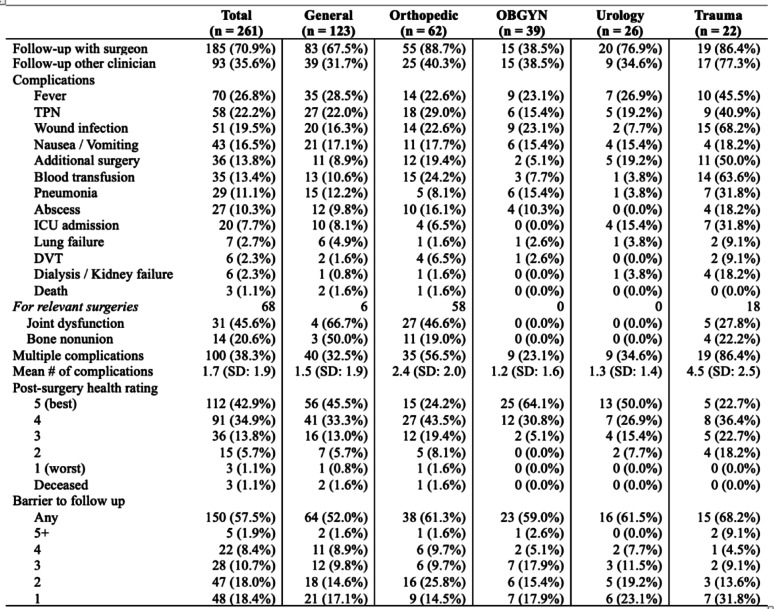
Patient postoperative complications, health outcomes, and follow-up characteristics by surgery type

Surgery type, complication type, and follow-up were not mutually exclusive categories, so totals may add to more than 261 or 100%. There were 7 other surgery types recorded that were not included in this table due to low numbers: vascular (*n* = 1), neonatal / pediatric (*n* = 4), ophthalmological (*n* = 1), spine (*n* = 1), and unknown (*n* = 2)

### Survey models

After accounting for hospital-level factors, trauma and orthopedic surgeries were significantly associated with elevated odds of postoperative complications (Table 2). Patients who underwent trauma surgery faced significantly higher odds of all assessed complications except fever and nausea or vomiting. The odds of experiencing any postoperative complication were five times higher for trauma surgery patients (5.33; 95% CI: 1.15–54.57), while the odds of experiencing multiple complications were even higher (6.80; 2.22–27.17). Trauma patients had more than ten times higher odds of requiring a blood transfusion compared to those undergoing other types of surgeries (10.08; 3.47–31.32). They also had 8.60 higher odds of developing a wound infection (3.07–25.87), and 5.97 higher odds of requiring additional surgery (2.05–17.84).

Orthopedic surgery also demonstrated notable increased complication risk. Patients who underwent orthopedic surgery had nearly three times higher odds (2.90; 1.09–8.70) of experiencing any complication than those who did not have orthopedic surgery. As with trauma patients, the odds of a blood transfusion were higher (2.98; 1.15–7.84). For general, obstetrics and gynecology, and urology-related surgeries, we found minimal, if any, significant associations between surgery type and complications. Patients who underwent general surgery had lower odds (0.35; 0.13–0.88) of requiring additional surgery compared to those who did not undergo general surgery. Patients who had urology surgery had 0.18 odds (0.02–0.77) of requiring a blood transfusion (Table 2).

**Table 2:** Odds ratios for postoperative complications by surgery type

Complication	Unadjusted		Adjusted	
	OR (95% CI)	*p*-value	OR (95% CI)	*p*-value
**General surgery**				
Any (≥ 1)	0.71 (0.42–1.20)	0.20	0.98 (0.47–2.05)	0.96
Multiple (≥ 2)	0.63 (0.38–1.04)	0.07	0.67 (0.34–1.30)	0.24
Fever	1.20 (0.69–2.07)	0.52	1.32 (0.63–2.78)	0.46
TPN	0.99 (0.55–1.76)	0.96	0.86 (0.39–1.87)	0.71
Wound infection	0.68 (0.36–1.26)	0.22	1.01 (0.42–2.39)	0.98
Nausea / vomiting	1.11 (0.58–2.14)	0.75	2.22 (0.94–5.28)	0.07
Additional surgery	0.46 (0.21–0.95)	0.03	0.35 (0.13–0.88)	0.03
Blood transfusion	0.64 (0.30–1.30)	0.22	0.82 (0.32–2.03)	0.66
**Orthopedic surgery**				
Any (≥ 1)	5.05 (2.28–13.10)	< 0.0001	2.90 (1.09–8.70)	0.03
Multiple (≥ 2)	2.65 (1.49–4.76)	0.0009	1.81 (0.84–3.99)	0.13
Fever	0.76 (0.38–1.45)	0.41	0.45 (0.17–1.12)	0.09
TPN	1.63 (0.85–3.09)	0.14	1.30 (0.52–3.16)	0.57
Wound infection	1.31 (0.64–2.56)	0.45	0.96 (0.35–2.52)	0.93
Nausea / vomiting	1.19 (0.54–2.44)	0.66	0.95 (0.35–2.48)	0.92
Additional surgery	1.79 (0.82–3.74)	0.14	1.46 (0.54–3.83)	0.45
Blood transfusion	2.89 (1.37–6.01)	0.006	2.98 (1.15–7.84)	0.02
**Obstetrics and gynecology surgery**				
Any (≥ 1)	0.57 (0.28–1.15)	0.11	0.71 (0.16–3.36)	0.65
Multiple (≥ 2)	0.45 (0.20–0.94)	0.03	0.83 (0.14–3.68)	0.81
Fever	0.80 (0.35–1.70)	0.57	2.39 (0.50–10.82)	0.26
TPN	0.61 (0.23–1.42)	0.26	1.45 (0.25–6.44)	0.65
Wound infection	1.30 (0.56–2.82)	0.53	1.04 (0.10–5.46)	0.97
Nausea / vomiting	0.92 (0.34–2.17)	0.85	0.43 (0.003–3.99)	0.52
Additional surgery	0.36 (0.07–1.14)	0.09	0.29 (0.002–2.78)	0.34
Blood transfusion	0.55 (0.14–1.57)	0.29	1.78 (0.17–10.78)	0.58
**Urology surgery**				
Any (≥ 1)	0.46 (0.21–1.06)	0.07	0.42 (0.17–1.03)	0.06
Multiple (≥ 2)	0.86 (0.36–1.93)	0.72	0.75 (0.30–1.80)	0.53
Fever	1.02 (0.39–2.40)	0.96	0.84 (0.31–2.09)	0.72
TPN	0.85 (0.29–2.14)	0.74	0.85 (0.28–2.29)	0.76
Wound infection	0.38 (0.07–1.22)	0.11	0.40 (0.07–1.44)	0.17
Nausea / vomiting	0.95 (0.29–2.57)	0.93	0.59 (0.17–1.73)	0.35
Additional surgery	1.64 (0.54–4.24)	0.36	1.30 (0.40–3.71)	0.65
Blood transfusion	0.34 (0.04–1.37)	0.15	0.18 (0.02–0.77)	0.02
**Trauma surgery**				
Any (≥ 1)	6.97 (1.74–63.41)	0.003	5.33 (1.15–54.57)	0.03
Multiple (≥ 2)	10.84 (3.77–41.84)	< 0.0001	6.80 (2.22–27.17)	0.0005
Fever	2.44 (1.00–5.83)	0.05	2.11 (0.77–5.67)	0.14
TPN	2.86 (1.14–7.02)	0.03	3.70 (1.24–11.40)	0.02
Wound infection	11.35 (4.56–30.65)	< 0.0001	8.60 (3.07–25.87)	< 0.0001
Nausea / vomiting	1.26 (0.37–3.49)	0.68	1.10 (0.30–3.43)	0.88
Additional surgery	8.29 (3.31–20.97)	< 0.0001	5.97 (2.05–17.84)	0.001
Blood transfusion	17.10 (6.72–46.28)	< 0.0001	10.08 (3.47–31.32)	< 0.0001

The adjusted models account for hospital-level factors

### Barriers to follow-up care

Barriers to seeking follow-up care were reported by 57.5% of participants (150/261), as shown in Table 1. Patients reported a mean of 1.3 barriers (range: 0–6), with 39.1% (102/261) experiencing multiple. Transportation (36.0%, 94/261), cost (31.8%, 83/261), appointment availability (20.7%, 54/261), and proximity (19.9%, 52/261) were the most prevalent barriers. Reported barriers varied by surgery type: 68.2% of patients who received trauma surgery reported at least one barrier (15/22; x̅=1.5 barriers), followed by orthopedic (61.3%, 38/62; x̅=1.4), urology (61.5%, 16/26; x̅=1.3), and obstetrics and gynecology (59.0%, 23/39; x̅=1.4) patients (Table 3). These barriers were consistent with workshop accounts of transport insecurity, financial constraints, weak referral pathways, and limited service availability, which together hinder continuity of postoperative care.

**Table 3:**
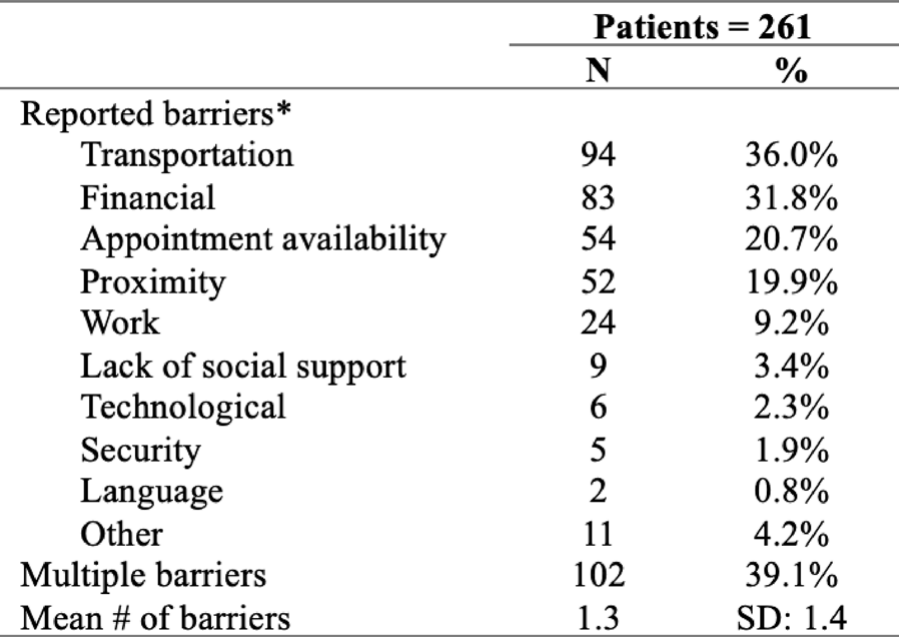
Barriers to follow-up care reported by patients

### Workshop findings

Findings from the workshops are reported by health system building block and summarized in Table 4. Workshop findings should be interpreted as stakeholder perspectives on factors perceived to shape postoperative care, rather than empirically tested predictors of complications.

- 1. Health workforce

The shortage of specialized surgeons was a significant concern in both workshop sessions. This deficit forces general surgeons to perform procedures outside their expertise. For instance, hospitals heavily affected by conflict often lack gynecologists and anesthesiologists, forcing surgeons to undertake procedures for which they lack formal training. This is exacerbated by the absence of a continuing medical education system, leaving many surgeons unprepared to handle the evolving demands of surgical care, without adequate training on the effective management of various injuries, and relying on outdated practices. Participants also noted inadequate infection prevention and control (IPC) due to insufficient training and lack of formal protocols; this was perceived as a likely contributor to postoperative complications. Lastly, high patient loads have made it nearly impossible to provide individualized care, with some doctors reportedly seeing up to 400 patients in a single shift.

- 2. Service delivery

Surgeons and administrators noted poor continuity of care, as many doctors work in multiple hospitals and often with inconsistent schedules, making it difficult for patients to receive follow-up care from the same surgeon. They noted that patients often live in remote locations or frequently relocate, which likewise hinders patients’ access to care. These findings aligned with survey results, in which patients reported that distance from health facilities and transportation-related barriers limited their ability to access care. Stakeholders identified the lack of standardized postoperative care protocols across hospitals as another major barrier to effective service delivery. Relatedly, surgeons and hospital administrators pointed to the limited education that patients receive at discharge as another challenge.

- 3. Health information systems

Each hospital represented in the workshop operated on different health information systems, making continuity of care between them challenging. Stakeholders mentioned that without a unified system, critical patient information such as surgical history, postoperative follow-up visits, and complication records are often lost or inaccessible, leading to inefficiencies and potential risks to the patient. This is especially relevant as patients often move between facilities for care.

- 4. Access to essential medicines and equipment

Participants highlighted challenges in accessing medicines and equipment, with NGOs noting that hospitals often lack sufficient medical supplies. When hospitals do receive new equipment, there is often no one available to conduct necessary training. Surgeons and hospital administrators emphasized that the conflict has made accessing essential medicines difficult, and NGO stakeholders raised concerns about the availability of expired or substandard medications.

- 5. Health financing

Limited and inconsistent funding was raised as a significant barrier to improving postoperative care by all participants. NGO stakeholders highlighted numerous challenges with a donor-driven funding model, where hospitals are pressured to meet unrealistic surgical quotas set by donors and will sometimes misreport procedures to secure funding. Hospital administrators and surgeons were frustrated by low and declining compensation rates, which make it difficult to retain staff. Likewise, patients’ financial constraints, such as difficulty affording transportation or essential medicines, negatively impact care.

- 6. Leadership and governance

Participants identified numerous governance challenges, particularly related to accountability and the enforcement of medical protocols. These challenges are compounded by the absence of a centralized health authority in northwest Syria. Furthermore, coordination between healthcare facilities and among medical personnel was noted as insufficient. NGO stakeholders advocated for governance structures that can consistently uphold standards across both public and private sectors.

**Table 4:** Health system challenges contributing to postoperative complications

Health system building block	Hospital administrators and surgeons	NGO stakeholders	Both
Health workforce	-Inadequate training on the effective management of injuries, particularly conflict-related injuries	-Shortage of specialized surgeons and other medical doctors	-Limited opportunities for continuing medical education and other training -Severe workforce shortages -Overworked health workers -Insufficient training on adherence to IPC protocols
Service delivery	-Surgeons rotating across hospitals with little coordination -Gaps in care and communication -Lack of standardized postoperative procedures -Limited patient education		
Health information systems	-Difficulties tracking patients across hospitals	-Underreporting of complications	-Absence of standardized medical records -Lack of a unified health information system across hospitals
Access to medicines and equipment		-Limited medical equipment and training on its use -Insufficient access to necessary medication	
Health financing	-Low or declining compensation rates leading -Financial constraints faced by patients	-Pressures to meet unrealistic surgical quotas - Demands of donor-driven funding model	-Inconsistent and insufficient funding for hospitals
Leadership and governance	-Insufficient coordination among medical personnel	-Lack of accountable leadership supporting oversight -Inconsistent enforcement of clinical protocols -Poor integration between public, private and humanitarian providers	

### Proposed interventions

**Fig. 1:**
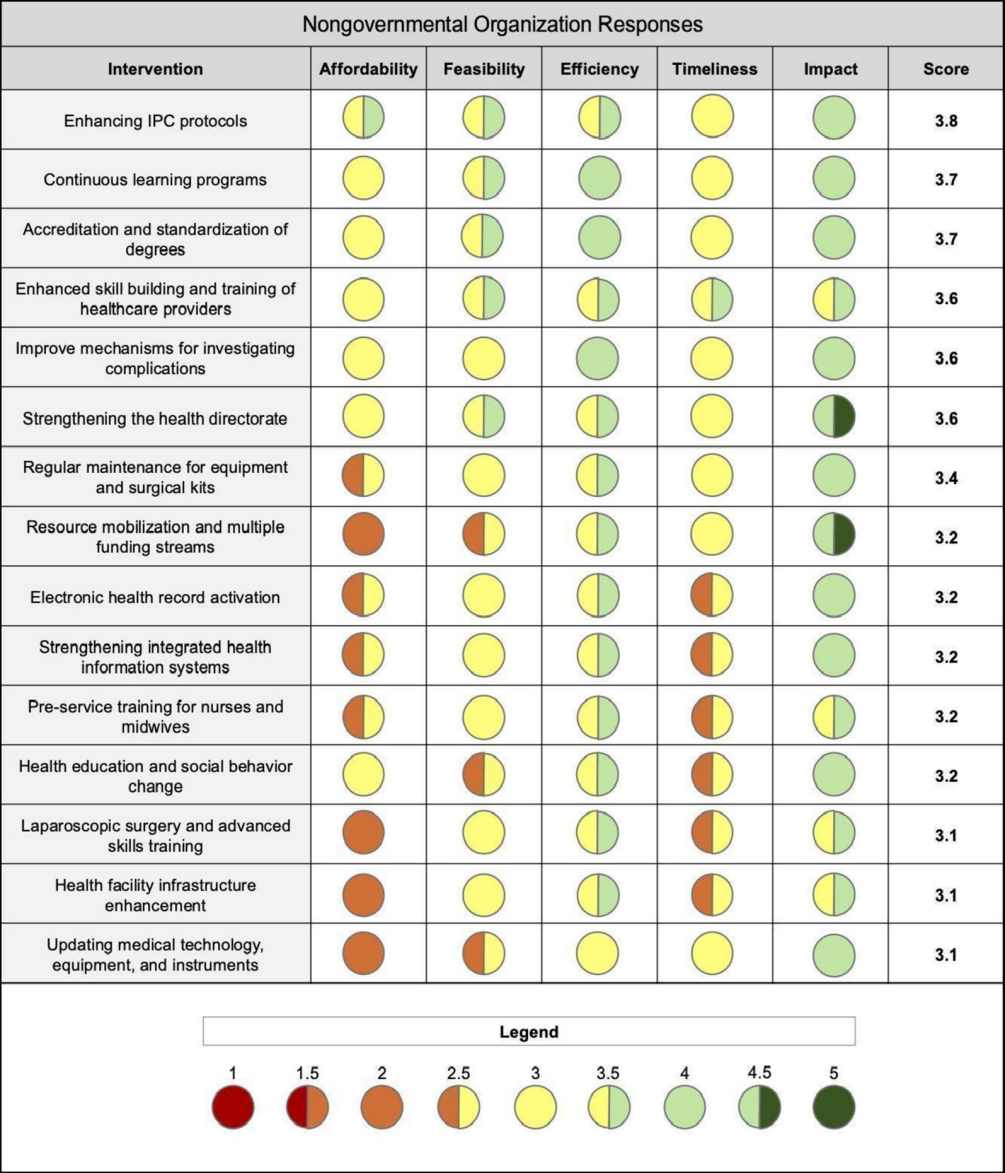
Prioritization of interventions identified by NGO stakeholders based on affordability, feasibility, efficiency, timeliness, and impact. Scores reflect the independent ratings of 13 NGO stakeholders. Overall scores represent the mean of individual ratings across all five evaluation criteria for each intervention; higher scores indicate higher priority

**Fig. 2:**
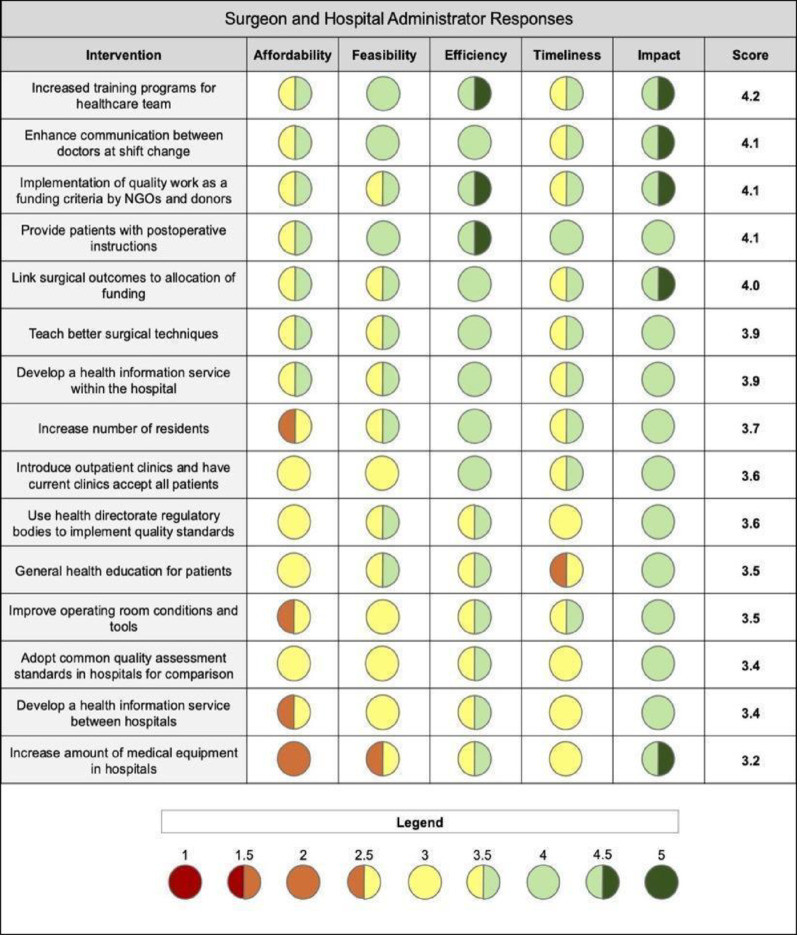
Prioritization of interventions identified by surgeons and hospital administrators based on affordability, feasibility, efficiency, timeliness, and impact. Scores reflect the independent ratings of 18 hospital surgeons and administrators. Overall scores represent the mean of individual ratings across all five evaluation criteria for each intervention; higher scores indicate higher priority

Figures 1 and 2 present the interventions identified and prioritized by participants in workshop sessions. NGO stakeholders prioritized increased training, as well as improved IPC practices and complication investigation (Fig. 1). Surgeons and hospital administrators likewise prioritized increased training, though also highlighted interventions focused on improving internal operations (e.g., communication at shift change and provision of discharge instructions) and that financially incentivize quality work (Fig. 2). Both groups proposed larger infrastructural interventions that were deemed high-impact but unlikely to be affordable or feasible.

## Discussion

This study offers a multifaceted perspective on the burden of postoperative complications in conflict-affected northwest Syria. Considerable research has documented the wide-ranging impacts of Syria’s healthcare crisis and how over a decade of armed conflict has profoundly deteriorated its health infrastructure [12–14]. Our findings illustrate how these systemic vulnerabilities shaped outcomes for surgical patients and the surgical ecosystem in northwest Syria. While limited research has examined postoperative complications and barriers to follow-up care at the patient and system level in northwest Syria, results from other hospital-based surgical and trauma care studies align with many of our findings [14–16].

### Patient-reported barriers and broader systemic gaps

Surgeons and hospital administrators noted that ensuring adherence to IPC practices could reduce overall treatment costs and improve outcomes, but efforts to ensure adherence are hindered by weak governance structures. This is exacerbated by informal workarounds that have emerged in response to chronic workforce shortages, lack of oversight, and operational instability.

Displacement and insecurity have severely constrained health-seeking behavior, particularly given the limited referral pathways and affordable access. This choice is reflected by Haar et al., who found that patients were often forced to choose between the threat of conflict-related violence at hospitals or the substantial financial burden of seeking health services at costlier private facilities [13].

Our findings underscore the critical shortage of surgeons, specialist doctors, and other health staff across hospitals in northwest Syria [13]. These conditions, compounded by widespread workforce exhaustion and pressures to meet surgical quotas, reflect a system operating beyond capacity and constraining patients’ ability to access timely follow-up care. Patients are also frequently discharged without clear guidance, including instructions on accessing follow-up care; this is less a result of negligence than of systemic pressures.

### Burden of postoperative complications by surgery type

In many conflict settings, fragile trauma systems are further undermined by the departure of health workers, disruptions to supply chains, and attacks on health infrastructure [17]. In northwest Syria, these challenges are particularly pronounced; the limited number of specialized doctors forces general surgeons to take on cases beyond their formal training, increasing the risk of adverse outcomes. These findings highlight how trauma and orthopedic patients in northwest Syria face compounding disadvantages from clinical complexity, structural barriers, and ongoing conflict, underscoring the need for targeted strategies to improve outcomes in this population.

### Fragmentation in health information systems

With parallel data systems, the lack of interoperability obstructs longitudinal patient tracking and limits the potential for quality improvement initiatives. Data collected in the region has been used primarily for advocacy and funding efforts rather than to inform healthcare provision and policy planning since the conflict began [18]. Participants consistently emphasized that without improvements in health information systems, even well-trained staff will struggle to coordinate care or identify trends in surgical outcomes.

### Cross-validation and integration of findings

The convergence across data sources indicates how structural and clinical barriers intersect to shape postoperative outcomes, suggesting that these outcomes are influenced not only by clinical factors but also by existing weaknesses explained by health workers and NGO stakeholders. This cross-validation is especially evident in the elevated complication rates reported among trauma and orthopedic surgery patients. These findings highlight the technical complexity of procedures and the chronic underinvestment in surgical specialization. They also reflect workshop concerns about limited specialized surgical capacity, inadequate equipment, and insufficient training on conflict-related injuries.

### Proposed interventions

The NGO stakeholders prioritized improving IPC (Fig. 1), reflecting a concern that wound infections may signal broader weaknesses in infection prevention and control practices. The convergence across all workshop participants on the need for continuing medical education (Figs. 1 and 2) underscores the importance of investing in workforce development during the health system’s rebuilding process. This includes establishing standardized accreditation mechanisms, which would enable the systematic training and credentialing of healthcare professionals in specialized surgical disciplines. Such a program could also expand the pool of qualified practitioners by creating a recognized and trusted pathway for developing expertise and specialized training. Participants noted that these structures could motivate recent medical graduates and junior clinicians to remain in Syria to pursue accredited specialization, helping to rebuild the country’s practitioner pool. In parallel, these programs could help standardize quality benchmarks across facilities and build trust among patients and NGOs, as well as improve professional mobility for health workers across different humanitarian and local governance networks.

Surgeons and hospital administrators advocated for enhancing communication between doctors during shift changes, given the role of ineffective patient handoffs in producing clinical errors and missed opportunities for follow-up care. Enhanced communication with patients at discharge, specifically regarding recognizing complications and accessing follow-up care, would likely reduce preventable morbidity and mortality. Additionally, aligning financial incentives for health facilities with clinical quality metrics rather than service volume was regarded as a powerful strategy to promote accountability and drive continuous improvement across surgical services. Such financial restructuring could also include implementing quality surgical outcomes as a funding criterion by NGOs and other donors. This could be an effective tool to shape how donors allocate funding across hospitals by rewarding institutions that demonstrate commitment to quality improvement.

### Limitations of the study

Several limitations should be considered. First, this study relied on self-reported complications and barriers from patients, which may be subject to recall bias or underreporting. Second, because the survey was conducted by phone, the sample may be subject to survivor and reachability bias, as patients who were reachable by phone and able to participate may have differed from those who could not be contacted. Third, the reported barriers are based on individual perceptions and may simplify or not fully capture the broader structural constraints that influence access and are less visible to patients. Responses were also not considered clinically validated outcomes or independently confirmed against clinical records, which increases the risk of misclassifying complications. Fourth, as this study used secondary survey data, recruitment flow information, including the number of patients unreachable by phone or who declined participation, was not available to the study team, precluding a full assessment of non-response bias. Finally, the findings are specific to conflict-affected northwest Syria and should not be assumed to apply to other parts of Syria or different settings with diverse health system structures.

## Conclusions

This study offers insights into the burden of postoperative complications and the barriers to surgical follow-up care in northwest Syria, a region where 13 years of armed conflict severely disrupted and fragmented the health system. We examined postoperative care at both the individual and system levels, drawing on patient-reported data alongside insights from frontline health workers and NGO stakeholders. The convergence between quantitative results and workshop findings suggests that systemic weaknesses, such as health workforce shortages, inadequate health information systems, limited opportunities for continuing medical education, and gaps in IPC, may be associated with patient-reported outcomes.

The proposed interventions can support the development of an accountable and adaptable surgical ecosystem as part of a more resilient health system. Postoperative care serves as a litmus test for health system resilience in fragile and conflict-affected settings, where the continuum of care is frequently interrupted. Priority areas for improvement include strengthening post-discharge follow-up systems, improving referral coordination, reinforcing IPC practices, and supporting the health workforce through, for example, training and credentialing mechanisms. We encourage future longitudinal studies that monitor postoperative outcomes over time and across facilities to further understand the cumulative impact of interventions.

## Data Availability

Data can be made available upon reasonable request.

## Abbreviations

AFAQ: Horizons for humanitarian relief organization
NGO: Non-governmental organization
WHO: World health organization
IPC: Infection prevention and control
NWS: Northwest Syria
